# *Histoplasma capsulatum* requires peroxisomes for multiple virulence functions including siderophore biosynthesis

**DOI:** 10.1128/mbio.03284-22

**Published:** 2023-07-11

**Authors:** Peter J. Brechting, Chandan Shah, Liva Rakotondraibe, Qian Shen, Chad A. Rappleye

**Affiliations:** 1 Department of Microbiology, Ohio State University, Columbus, Ohio, USA; 2 Division of Medicinal Chemistry and Pharmacognosy, College of Pharmacy, Ohio State University, Columbus, Ohio, USA; 3 Department of Biology, Rhodes College, Memphis, Tennessee, USA; The University of British Columbia, Vancouver, British Columbia, Canada

**Keywords:** Histoplasma, peroxisome, siderophores, virulence, macrophages, iron acquisition

## Abstract

Peroxisomes are versatile eukaryotic organelles essential for many functions in fungi, including fatty acid metabolism, reactive oxygen species detoxification, and secondary metabolite biosynthesis. A suite of Pex proteins (peroxins) maintains peroxisomes, while peroxisomal matrix enzymes execute peroxisome functions. Insertional mutagenesis identified peroxin genes as essential components supporting the intraphagosomal growth of the fungal pathogen *Histoplasma capsulatum*. Disruption of the peroxins Pex5, Pex10, or Pex33 in *H. capsulatum* prevented peroxisome import of proteins targeted to the organelle via the PTS1 pathway. This loss of peroxisome protein import limited *H. capsulatum* intracellular growth in macrophages and attenuated virulence in an acute histoplasmosis infection model. Interruption of the alternate PTS2 import pathway also attenuated *H. capsulatum* virulence, although only at later time points of infection. The Sid1 and Sid3 siderophore biosynthesis proteins contain a PTS1 peroxisome import signal and localize to the *H. capsulatum* peroxisome. Loss of either the PTS1 or PTS2 peroxisome import pathway impaired siderophore production and iron acquisition in *H. capsulatum*, demonstrating compartmentalization of at least some biosynthetic steps for hydroxamate siderophore biosynthesis. However, the loss of PTS1-based peroxisome import caused earlier virulence attenuation than either the loss of PTS2-based protein import or the loss of siderophore biosynthesis, indicating additional PTS1-dependent peroxisomal functions are important for *H. capsulatum* virulence. Furthermore, disruption of the Pex11 peroxin also attenuated *H. capsulatum* virulence independently of peroxisomal protein import and siderophore biosynthesis. These findings demonstrate peroxisomes contribute to *H. capsulatum* pathogenesis by facilitating siderophore biosynthesis and another unidentified role(s) for the organelle during fungal virulence.

**IMPORTANCE:**

The fungal pathogen *Histoplasma capsulatum* infects host phagocytes and establishes a replication-permissive niche within the cells. To do so, *H. capsulatum* overcomes and subverts antifungal defense mechanisms which include the limitation of essential micronutrients. *H. capsulatum* replication within host cells requires multiple distinct functions of the fungal peroxisome organelle. These peroxisomal functions contribute to *H. capsulatum* pathogenesis at different times during infection and include peroxisome-dependent biosynthesis of iron-scavenging siderophores to enable fungal proliferation, particularly after activation of cell-mediated immunity. The multiple essential roles of fungal peroxisomes reveal this organelle as a potential but untapped target for the development of therapeutics.

## INTRODUCTION

*Histoplasma capsulatum* is a primary fungal pathogen of mammals that survives in the phagosomal compartment of phagocytes. A number of mechanisms facilitate *H. capsulatum* infection of these host cells (1 - 3), and the host cell phagosome is initially permissive for *H. capsulatum* survival and proliferation until activation of phagocytes during the adaptive immune response (4 - 8). The mechanisms enabling *H. capsulatum* intracellular proliferation are only partly known, but the isolation of mutants able to infect, but unable to proliferate in phagocytes is revealing cellular processes essential for *H. capsulatum* intracellular proliferation. These processes include the metabolism of non-hexose substrates (9) and overcoming nutritional immunity through the acquisition of the micronutrients iron, copper, and zinc (4, 10 - 12). Compartmentalization of many reactions and biosynthetic steps is common in eukaryotic cells and is likely important for cellular functions of *H. capsulatum* involved in its pathogenesis of phagocytes.

Peroxisomes are organelles found in nearly all eukaryotic cells, and canonically house enzymes responsible for cellular functions such as the β-oxidation of fatty acids, steps of the glyoxylate metabolic pathway, and degradation of metabolically derived reactive oxygen species (ROS) (13 - 16). In addition, fungal peroxisomes are involved in the production of diverse secondary metabolites (17 - 20), vitamins (21 - 23), as well as iron-scavenging siderophores (24). The maintenance of peroxisomes is mediated by a suite of over 30 Pex proteins dubbed peroxins (25, 26). These peroxins play crucial roles in the biogenesis of peroxisomes and also mediate peroxisome function by enabling the selective import of peroxisome-targeted proteins into the lumen of the organelle (26).

Peroxisome-targeted proteins contain one of two peroxisome-targeting sequences: a *C*-terminal three amino acid peroxisome-targeting signal (PTS1) or a less conserved *N*-terminal signal (PTS2). Peroxisome matrix proteins are captured from the cytosol by Pex5 or Pex7, which function as the cytosolic receptors for PTS1- and PTS2-containing proteins, respectively (27, 28). Pex5 and Pex7 shuttle matrix proteins to the peroxisomal docking complex, which consists of Pex13 and Pex14, along with Pex17 in some yeasts (25, 29). In *Neurospora crassa*, the Pex14-related protein Pex33 functions in place of Pex17 in this docking complex (30, 31). The docking complex translocates the PTS-containing protein into the organellar lumen (29, 32), and a RING-finger complex consisting of Pex2, Pex10, and Pex12 recycles the Pex5 protein back to the cytosol through a mono-ubiquitination process (33 - 35). While the mechanism of Pex7 recycling in yeast has not been fully characterized, this process does appear to interact with Pex2, Pex10, and Pex12-based recycling of Pex5 in yeast (36). The concerted action of these peroxins results in the localization of matrix proteins within the peroxisome, which defines the functions executed by the organelle.

Some virulence functions of pathogenic yeasts have been linked to peroxisomes in diverse hosts. In the plant pathogen *Alternaria alternata*, disr*Alternaria alternata*uption of peroxisome function prevents the formation of appressorium-like structures and toxin production, which impairs the virulence of citrus plant leaves (20, 22, 23). In the cereal crop pathogen *Fusarium graminearum*, peroxisome function is required for ROS tolerance, toxin production, and full pathogenicity (37). Peroxisomes also contribute to the virulence of fungal pathogens during mammalian infections, largely due to their role in the compartmentalization of metabolic pathways. For example, the peroxisomal β-oxidation pathway in *Cryptococcus neoformans* is required for full virulence of yeasts, likely due to energy requirements for stress response and capsule formation (38). In the opportunistic pathogen *Candida albicans*, peroxisomal enzymes mediate the glyoxylate cycle, which is necessary for virulence; however, mislocalization of these enzymes to the cytosol did not recapitulate the attenuated virulence, leaving the essentiality of peroxisomes in this process unclear (39, 40). In the pathogen *Candida glabrata*, autophagic degradation of peroxisomes facilitates fungal survival during infection of mammalian cells *in vitro*, possibly as a method of nutrient recycling (41). However, these diverse fungal mechanisms connected to peroxisomes are unlikely to contribute to *H. capsulatum* pathogenesis since *H. capsulatum* cells do not form appressoria or capsules, the glyoxylate cycle enzyme isocitrate lyase (Icl1) is dispensable for *H. capsulatum* virulence (9), and the peroxisomal catalase (CatP) alone is not required for *H. capsulatum* defense against phagocyte ROS (42).

In this study, genes identified in a forward genetic screen for mutants of *H. capsulatum* that exhibited attenuated intracellular proliferation in phagocytes (4) highlight the essential role of peroxisomes for *H. capsulatum* intracellular growth. We discovered that disruption of both the PTS1 and PTS2 peroxisomal matrix protein import pathways impairs *H. capsulatum* virulence, although at different stages of infection, suggesting multiple peroxisome functions are required for *H. capsulatum* pathogenesis. Peroxin mutant characterizations demonstrate that peroxisomes contribute to *H. capsulatum* siderophore biosynthesis and reveal the existence of another peroxisome function controlled by the PTS1 peroxisome protein import pathway.

## RESULTS

### *H. capsulatum* intramacrophage growth and virulence require peroxins

To identify *H. capsulatum* genes required for intracellular replication in macrophages, we screened insertion mutants of *H. capsulatum* for mutants with impaired intracellular growth (4). Insertional mutants were generated using *Agrobacterium tumefaciens*-mediated transformation of a T-DNA element, and the intracellular growth of *H. capsulatum* was monitored directly by fluorescence using a strain of *H. capsulatum* expressing td-Tomato RFP or indirectly by measuring macrophage lysis that occurs following intracellular fungal replication. Mutants were selected that showed less than a 30% increase in RFP fluorescence over time and/or at least 30% reduction in macrophage lysis.

Three T-DNA insertions associated with attenuated *H. capsulatum* growth in macrophages mapped to genes encoding proteins homologous to the peroxins Pex5, Pex10, and Pex33. The disrupted *H. capsulatum* genes in mutants 79C10 and 07A9 were designated the orthologs of genes encoding Pex5 and Pex10, respectively, based on reciprocal top-hit BLAST of Pex5 and Pex10 from *Saccharomyces cerevisiae*. The T-DNA insertion in 79C10 was located within the third exon of the *PEX5* gene (998 nucleotides downstream of the CDS start) and the insertion in 07A9 disrupted the first exon of *PEX10* (82 nucleotides downstream of the CDS start). Although the *H. capsulatum* Pex33 protein shows homology to Pex14 of *S. cerevisiae*, the reciprocal top-hit BLAST search of *N. crassa* indicated that the gene mutated in mutant 502F2 encodes a protein orthologous to Pex33. The T-DNA insertion in mutant 502F2 mapped to the promoter region of *PEX33* (168 bp upstream of the start codon). These mutations are predicted to cause loss of peroxin functions either through disruption of the coding sequence (79C10 and 07A9) or interference with peroxin-encoding gene transcription (502F2).

**Fig 1:**
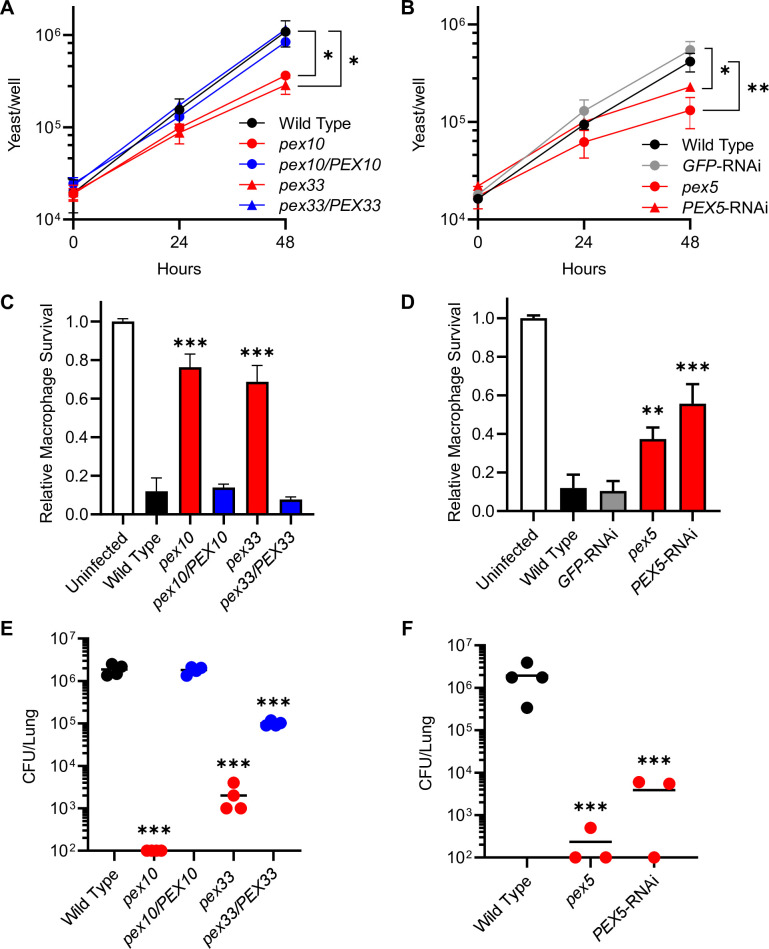
Intramacrophage growth and virulence of *H. capsulatum* require peroxisome function. *H. capsulatum* intracellular growth (**A and B**), virulence in macrophages (**C and D**), and virulence in murine infection (**E and F**) were tested for peroxin-deficient yeasts. Peroxin-deficient yeasts (red symbols), either *pex10*, *pex33*, *pex5* insertional mutants, or RNAi-depleted strain (*PEX5*-RNAi), were compared to wild-type (black) and complemented strains (blue) or to a control RNAi strain (*GFP*-RNAi; gray). (**A and B**) Proliferation of yeasts in macrophages was quantified by counting yeast cells in macrophage lysates by hemacytometer with the determination of viability by staining with the Hoechst 33342 dye. Data points represent the mean number of viable yeasts (± SD) from biological replicate infections (*n* = 3). (**C and D**) As a measure of *H. capsulatum* virulence, survival of *H. capsulatum*-infected cultured macrophages after 7 days of infection was measured by quantifying the remaining macrophage-expressed β-galactosidase activity. Data represent the mean survival (± SD) of macrophages following infection with biological replicates (*n* = 3). (**E and F**) The virulence of peroxin-deficient yeasts *in vivo* was determined by quantifying the fungal burden of *H. capsulatum* (CFUs) in lungs 8 days following intranasal infection of mice with 3 × 10^4^ yeasts. Individual data points are shown (*n* = 3–4 mice) with horizontal bars representing the mean fungal burden in the lungs. Asterisks indicate significant differences compared to the wild type by Student’s *t*-test (*P < 0.05, **P < 0.01, ***P < 0.001).

The independent identification of multiple peroxin-encoding genes strongly suggests that *H. capsulatum* requires peroxisome function(s) for the pathogenesis of macrophages. To directly determine the consequence of loss of peroxin functions on intracellular proliferation, macrophages were infected with each mutant and the number of viable cells was determined. Early experiments failed to recover any viable CFUs from plating macrophage lysates on solid media, the reasons for which remain unclear. Consequently, to measure intracellular proliferation, macrophages were lysed and the number of yeasts was determined by counting yeasts directly by hemacytometer and scoring yeast viability using the membrane impermeant Hoechst 33342 dye. The vast majority of recovered yeasts, both wild type and mutant, were viable in macrophages, but the *pex10* and *pex33* mutants did not proliferate as well as wild-type *H. capsulatum* (Fig. 1A). Complementation of the *pex10* and *pex33* mutants restored their proliferation ability, confirming the decreased intracellular proliferation was linked to the loss of Pex10 and Pex33. Loss of Pex5 function similarly decreased intracellular proliferation (Fig. 1B). Independent depletion of Pex5 through RNAi of *PEX5* phenocopied the intracellular proliferation defect of the *pex5* mutant, demonstrating that the decreased growth was a consequence of the loss of Pex5. Impaired intracellular growth on the loss of Pex5, Pex10, and Pex33 protein functions strongly attenuated the ability of *H. capsulatum* to cause the death of host macrophages (Fig. 1C and D), and this attenuation was restored by *PEX10* and *PEX33* expression in *pex10* and *pex33* mutants (Fig. 1C) or was similarly impaired by RNAi-based depletion of *PEX5* expression (Fig. 1D).

To determine the necessity of *H. capsulatum* peroxisome function on pathogenesis *in vivo*, the peroxin mutants were tested in a murine respiratory model of infection. After 8 days of infection, the loss of *pex5*, *pex10*, or *pex33* severely reduced the fungal burden in the lungs as determined by the recovery of CFUs (Fig. 1E and F). Given the difficulty in recovering CFUs of *pex5*, *pex10*, and *pex33* mutants from macrophage lysates, the decreased fungal burden in the lungs was independently confirmed by quantitative PCR (qPCR) quantification of fungal genomes in the lung homogenates and by histology of lung sections (GMS [Grocott's methenamine silver]-stained yeasts). Depletion of *PEX5* (*PEX5*-RNAi) reduced *H. capsulatum* yeast proliferation in the lungs by 14-fold and 10-fold as measured by the number of fungal genomes within the lungs (Fig. S1A) or by the number of yeast cells in lung sections (Fig. S1B and C), respectively. These data indicate the essentiality of Pex5, Pex10, and Pex33 for the virulence of *H. capsulatum*.

### Pex5, Pex10, and Pex33 facilitate peroxisomal matrix protein import in *H. capsulatum*

**Fig 2:**
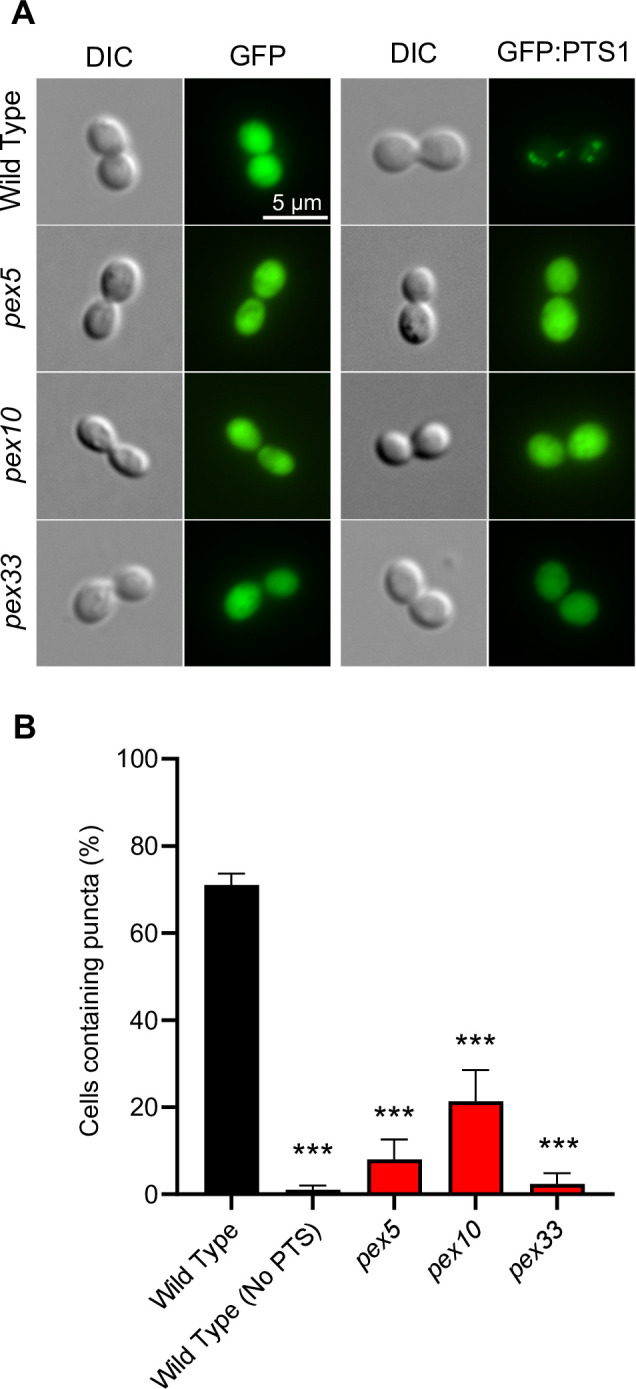
PTS1-dependent import of peroxisome-targeted proteins requires Pex5, Pex10, and Pex33. Localization of peroxisome-targeted GFP was determined by microscopy for wild-type *H. capsulatum* yeasts and Pex5-, Pex10-, and Pex33-deficient yeasts. (**A**) Differential interference contrast (DIC) and fluorescence microscopy images show the localization of GFP or GFP targeted to the peroxisome by transcriptional fusion of a PTS1 sequence to the *C*-terminus (GFP:PTS1). GFP or GFP:PTS1 was expressed in wild-type and Pex5-, Pex10-, or Pex33-deficient yeasts. Scale bar represents 5 µm. (**B**) Peroxisomal localization of peroxisome-targeted GFP was determined by quantifying the localization of the GFP into subcellular puncta in wild-type and peroxin-deficient strains. The peroxisome-targeted GFP (GFP:PTS1) was expressed in wild-type cells (Wild Type) or peroxin-deficient yeasts (*pex5*, *pex10*, and *pex33*), and the number of yeast cells in the population with GFP fluorescence localized to one or more subcellular puncta were quantified. Wild-type cells expressing GFP lacking the PTS1 signal [Wild Type (No PTS)] served as the background control. Data represent the average (± SD) of biological replicates (*n* = 3) with 150 yeasts cells scored per replicate for each strain. Asterisks indicate significant differences compared to the wild type by Student’s *t*-test (***P < 0.001).

Previous studies in fungi have demonstrated roles for Pex5, Pex10, and Pex33 in the import of proteins which contain one of two peroxisomal targeting sequences, PTS1 or PTS2, into the peroxisomal matrix. Pex5 acts specifically as a cytosolic receptor for proteins in the cytosol that contain the *C*-terminal PTS1 (43, 44), while Pex33 functions as a member of the protein translocon that imports PTS-containing proteins across the peroxisomal membrane (30). Pex10 functions in the ubiquitination of Pex5 to initiate its recycling back to the cytosol (34). To test if *H. capsulatum* Pex5, Pex33, and Pex10 contribute to the peroxisomal import of PTS1-containing proteins, the import of a PTS1-tagged GFP (GFP:PTS1) in *pex5*, *pex33*, and *pex10* mutants was assessed. For peroxisomal targeting of GFP, the *C*-terminal three amino acids from the peroxisomal catalase protein (CatP) that includes a recognizable PTS1 sequence (PRL) were fused to the *C*-terminus of GFP. In wild-type *H. capsulatum*, the GFP:PTS1 fusion protein localized to multiple puncta consistent with subcellular organelles (Fig. 2A). The fluorescent puncta do not co-localize with nuclei, endoplasmic reticulum, or mitochondria (Fig. S2). The localization of GFP depended on the *C*-terminal PTS1 sequence, as GFP lacking the PTS1 signal showed diffuse cytosolic fluorescence (Fig. 2A). Loss of Pex5, Pex10, or Pex33 function prevented the localization of the GFP:PTS1 to puncta (Fig. 2A and B), while complementation of Pex10 and Pex33 fully restored the punctate localization (Fig. S3). The dependence of the discrete subcellular compartmentalization of fluorescence on both the presence of a PTS1 sequence and peroxin functions indicates the puncta represent peroxisomes, and the import of the GFP:PTS1 into peroxisomes requires the PTS1-protein import pathway that includes Pex5, Pex33, and Pex10.

### PTS2 protein import pathway is necessary for *H. capsulatum* virulence only during adaptive immunity

**Fig 3:**
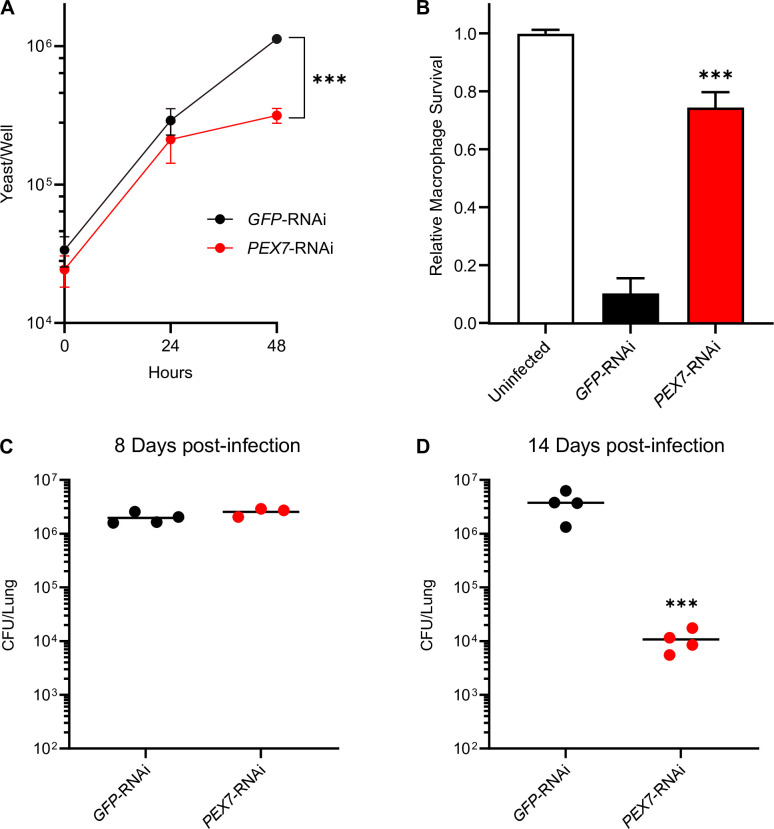
The PTS2 protein import pathway is necessary for *H. capsulatum* virulence during adaptive immunity. *H. capsulatum* intracellular growth (**A**), virulence in macrophages (**B**), and virulence in murine infection (**C and D**) were tested for Pex7-deficient yeasts (*PEX7*-RNAi) and compared to Pex7-expressing yeasts (*GFP*-RNAi). (**A**) Proliferation of yeasts in macrophages was quantified by measurement of intracellular yeasts by counting yeast cells in macrophage lysates by hemacytometer and determination of viability by staining with the Hoechst 33342 dye. Data points represent the mean number of viable yeasts (± SD) from biological replicate infections (*n* = 3). (**B**) Survival of *H. capsulatum*-infected macrophages was quantified after 7 days of infection by measurement of the remaining macrophage-derived β-galactosidase activity. Data points represent the mean surviving macrophage population (± SD) from biological replicate infections (*n* = 3). (**C and D**) The virulence of Pex7-deficient yeasts *in vivo* was determined by quantifying the fungal burden (CFU) of *H. capsulatum* in lungs 8 days (**C**) or 14 days (**D**) following intranasal infection of mice with 3 × 10^4^ yeasts. Individual data points are shown (*n* = 4 mice) with horizontal bars representing the mean fungal burden in lungs. Asterisks indicate significant differences compared to the wild type by Student’s *t*-test (***P < 0.001).

While the phenotypes of Pex5-deficient strains establish the role of PTS1-protein import in *H. capsulatum* virulence, they do not address the potential role of the PTS2-protein import pathway. To specifically examine the import of PTS2-containing proteins, we depleted the PTS2 cytosolic receptor, Pex7. The *H. capsulatum* Pex7 ortholog was identified by TBLASTN search of the *H. capsulatum* genome with the *S. cerevisiae* Pex7 protein. Depletion of Pex7 by RNAi impaired the intracellular proliferation of *H. capsulatum* yeasts as measured by the recovery of viable cells from infected macrophages (Fig. 3A) and attenuated the ability of yeasts to cause macrophage death (Fig. 3B) similar to the loss of PTS1-protein import machinery (Fig. 1). Depletion of Pex7 function did not cause a reduction in the lung fungal burden compared to Pex7-producing *H. capsulatum* yeasts 8 days post-infection of mice (Fig. 3C) unlike the loss of PTS1-dependent protein import (Fig. 1E and F). However, at 14 days post-infection, yeasts lacking Pex7 had lower lung fungal burdens compared to Pex7-producing yeasts (Fig. 3D). Depletion of Pex7 did not result in a plating defect, further differentiating the PTS1 and PTS2 pathways in *H. capsulatum*. These data show that the PTS2-protein import pathway is also required for full *H. capsulatum* yeast virulence, but that the PTS2-protein import pathway plays a distinct role from the PTS1-protein import pathway, specifically during later stages of infection.

### *H. capsulatum* peroxisome function is required for siderophore production and growth in limited iron

The requirement for the PTS1-protein import pathway for *H. capsulatum* virulence indicates that one or more PTS1-containing proteins contribute to the pathogenesis of *H. capsulatum* yeasts. Bioinformatic search of the *H. capsulatum* predicted proteome for PTS1-containing proteins using a consensus PTS1 sequence [A/S/C/P]-[K/R/H]-L (45) yielded 76 proteins (Table S1). Several proteins were homologous to fungal proteins with known or predicted functions (Table 1), including multiple proteins that are canonically localized to the peroxisome (e.g., CatP, peroxisomal catalase; Fox1, fatty acid oxidase; Mls1, malate synthase). Previous studies have shown that CatP, Fox1, and Icl1 are dispensable for *H. capsulatum* virulence (9, 42), removing catalase function, peroxisomal lipid catabolism, and the glyoxylate cycle from consideration as pathogenesis-enabling peroxisome functions.

**TABLE 1:** Predicted *H. capsulatum* proteins with canonical PTS1 sequences and predicted functions

Gene product	C-terminal sequence	Annotation	Top BLAST hit^*a*^
00600	SRL	Isocitrate dehydrogenase	AnIdh2 (XP_050469089.1)
00703	AKL	d-amino acid oxidase	AfDAO (XP_753520.1)
01285	AKL	Carnitine *O*-acetyltransferase	AfAcuJ (XP_663883.2)
01710	SKL	Mevalonyl-CoA hydratase	AfSidH (XP_748661.1)
02082 (Fox1)	SKL	Acyl-CoA oxidase	AnAoxA (XP_664356.1)
04029	AKL	Medium-chain fatty acid-CoA ligase	AnFaaB (XP_050467298.1)
04595	SHL	Siderochrome-iron transporter	AfMirC (XP_749702.2)
04681	AKL	NADP-dependent malic enzyme	AfMaeA (XP_755161.1)
04960 (Sid3)	SKL	Hydroxyornithine transacylase	AnSidF (XP_663838.1)
04963 (Sid1)	ARL	l-ornithine N5-oxygenase	AfSidA (XP_755103.1)
05832	SRL	LON domain serine protease	AfLON (XP_753475.1)
05934	SRL	Woronin body protein	AfHexA (XP_753754.2)
06870 (CatP)	PRL	Catalase	AnCatC (XP_663522.1)
08165 (Mls1)	AKL	Malate synthase	AfAcuE (XP_747723.1)

^*a*^ *Af* = *Aspergillus fumigatus* Af293, *An* = *Aspergillus nidulans* FGSC A4. Annotations were assigned by BLAST hits to *A. fumigatus* AF293, A*spergillus nidulans* FGSC A4, *Saccharomyces cerevisiae* S288c, *Schizosaccharomyces pombe* 972h, and *Neurospora crassa* OR74A.

**Fig 4:**
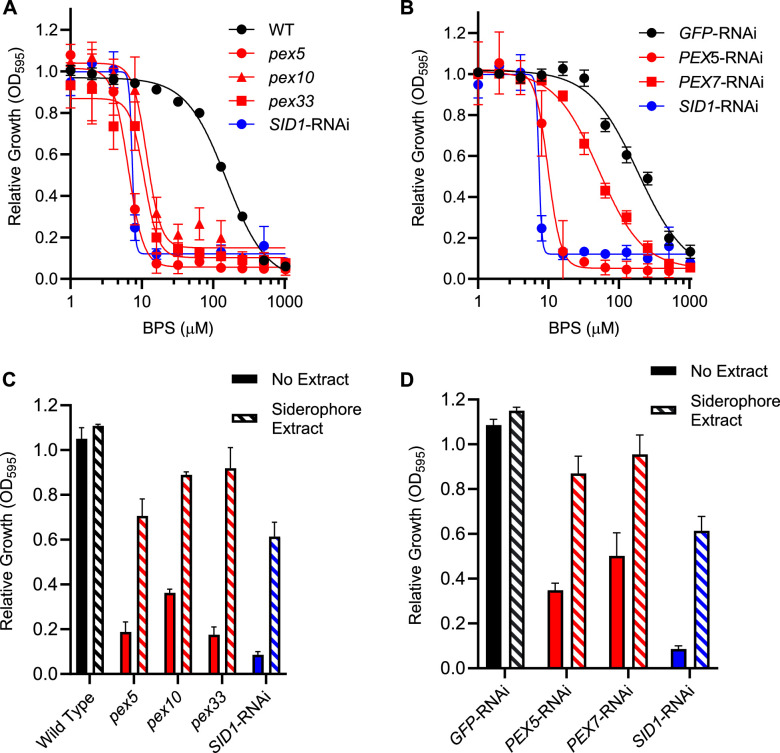
*H. capsulatum* peroxins are required for growth in low iron and siderophore production. Growth (optical density at 595 nm) of *H. capsulatum* peroxin-deficient yeasts (either mutant *pex* alleles or Pex-depletion through RNAi; red) was measured after 72 hours in restricted iron (**A and B**) or following siderophore supplementation (**C and D**) and compared to wild type (WT; black) or siderophore-deficient (*SID1*-RNAi; blue) yeasts. (**A and B**) Dose-response of *H. capsulatum* yeast growth in graded concentrations of the iron chelator BPS. Data points indicate the mean growth (± SD) of yeasts at each concentration of BPS normalized to growth with no chelator added. Curves represent a four-parameter non-linear regression fit to the data. (**C and D**) Rescue of peroxin-deficient growth in limited iron by exogenous siderophore was determined by growing yeasts in 20 µM BPS with 0.25% (vol/vol) water (No Extract; solid bars) or in 20 µM BPS with 0.25% (vol/vol) of a siderophore extract (Siderophore extract; hatched bars). The growth of yeasts was normalized to the growth of each strain in standard HMM media (contains 3 µM FeSO_4_), and the data represent the average relative growth (± SD) of biological replicates (*n* = 3).

Among the candidates in the PTS1-protein data set, two proteins (Sid1 and Sid3) from the *H. capsulatum* siderophore biosynthesis cluster (10) focused attention on the potential siderophore biosynthesis function of the *H. capsulatum* peroxisome. Studies in multiple fungi have established the role of siderophores in fungal acquisition of limited iron (23, 24, 46 - 49). To determine if peroxin mutants had decreased siderophore production, we tested the growth of peroxin-lacking *H. capsulatum* yeasts in limited iron. Wild-type *H. capsulatum* yeasts can grow in media containing the iron-chelator bathophenanthrolinedisulfonate (BPS) with an inhibitory concentration for 50% growth (IC_50_) of 152 µM, whereas yeasts unable to synthesize siderophores due to the depletion of Sid1 (*SID1*-RNAi) show 20-fold greater sensitivity to BPS restriction of iron (IC_50_ approximately 7 µM; Fig. 4A). Loss of PTS1-protein import without Pex5, Pex10, and Pex33 functions resulted in nearly identical inhibition of *H. capsulatum* growth by BPS as depletion of Sid1, indicating decreased ability to acquire limited iron (Fig. 4A). Depletion of the PTS2-protein import pathway (*PEX7-*RNAi) showed an intermediate sensitivity to BPS (IC_50_ = 51 µM) between wild-type and Sid1-depleted yeasts (Fig. 4B), demonstrating differences in the roles of PTS1- and PTS2-protein import pathways in the acquisition of limited iron.

To ensure that the inability of the peroxin mutants to grow in low-iron-containing medium resulted from deficient siderophore production, the ability of exogenous siderophores to restore growth in BPS-treated medium was tested. Culture filtrate extracts were collected from wild-type *H. capsulatum* yeasts grown in siderophore-inducing conditions (trace iron) and siderophore-repressing conditions (medium supplemented with 5 µM FeSO_4_). Siderophores were extracted from culture filtrates via diaion resin extraction, and fractions containing siderophore activity (based on the Chrome Azurol S chromogenic assay) were collected. The siderophore activity-containing fraction (Fig. S4A) from the low-iron medium culture filtrate restored the growth of the siderophore-deficient yeasts (*SID1*-RNAi) in iron-limiting conditions (20 µM BPS) in a dose-dependent manner (Fig. S4B), confirming the fraction contained functional siderophore molecules. Addition of 0.25% (vol/vol) of this siderophore-containing fraction to media rescued the growth of the peroxin mutant strains in iron-limiting conditions, restoring the growth of each strain between 60% and 95% of wild-type levels (Fig. 4C). A similar extract fraction from yeasts grown in siderophore-repressing conditions did not rescue the growth of Sid1-deficient yeasts (Fig. S4B). The siderophore-containing extract also restored growth of the PTS2-protein import-deficient strain (*PEX7*-RNAi) in an iron-limited medium (Fig. 4D). Complementation of Pex10 and Pex33 deficiencies also restored growth without siderophore extract supplementation, confirming the failure to grow was due to peroxisome deficiency (Fig. S4C). Together, these data show that *H. capsulatum* PTS1- and PTS2-protein import machinery are required for siderophore production and growth in iron-limiting conditions.

### *H. capsulatum* Sid1 and Sid3 localize to peroxisomes

**Fig 5:**
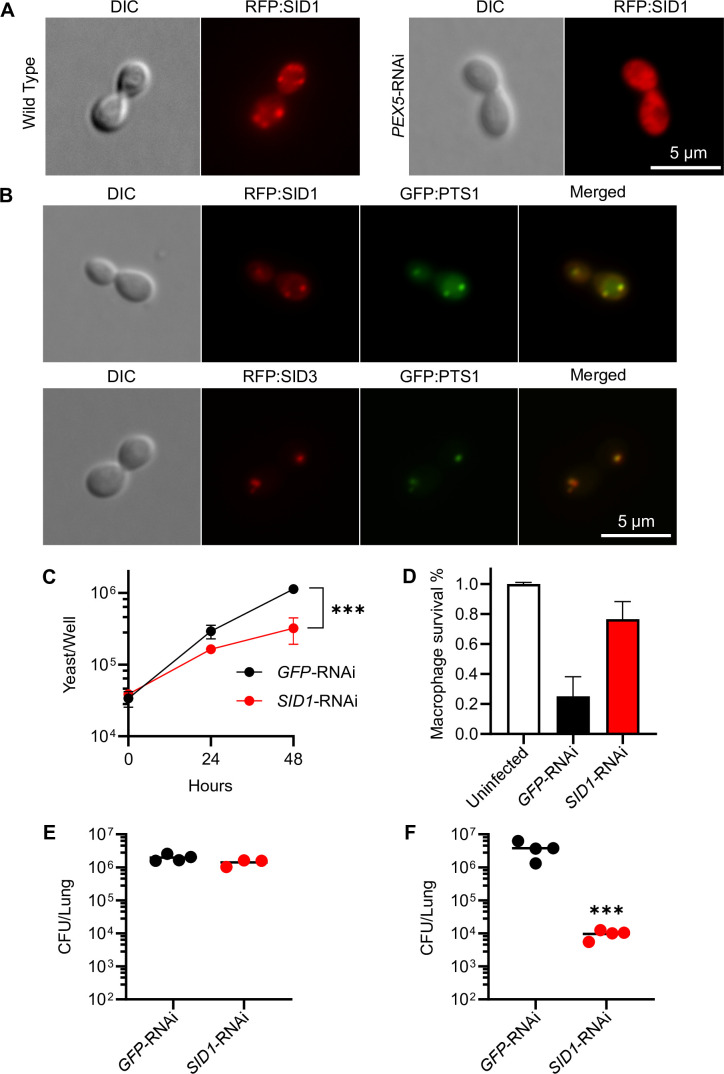
*H. capsulatum* Sid1 and Sid3 localize to peroxisomes, and siderophores are required for virulence only during adaptive immunity. The subcellular localization of *H. capsulatum* Sid1 and Sid3 proteins was determined by microscopy (**A and B**) and the contributions of Sid1 to *H. capsulatum* intracellular growth (**C**), virulence in cultured macrophages (**D**) and *in vivo* (**E**) were determined. (**A**) Differential interference contrast (DIC) and fluorescence microscopy images show the localization of an *N*-terminal translational fusion of RFP to Sid1 (RFP:Sid1) in wild-type and Pex5-deficient (PEX5-RNAi) yeasts. Scale bar represents 5 µm. (**B**) Microscopy images show the localization of Sid1 (RFP:Sid1) or Sid3 (RFP:Sid3) and the localization of peroxisome-targeted GFP (GFP:PTS1) to subcellular puncta in wild-type *H. capsulatum* yeasts. (**C**) Proliferation of yeasts in macrophages was quantified by measurement of intracellular yeasts by counting yeast cells in macrophage lysates by hemcytometer and determination of viability by staining with the Hoechst 33342 dye. Data points represent the mean number of viable yeasts (± SD) from biological replicate infections (*n* = 3). (**D**) Survival of *H. capsulatum-*infected macrophages was quantified after 7 days of infection by measurement of the remaining macrophage-derived β-galactosidase activity. Data represent the surviving macrophages (± SD) from biological replicate infections (*n* = 3). (**E and F**) The virulence of Sid1-deficient yeasts *in vivo* was determined by quantifying the fungal burden (CFU) of *H. capsulatum* in lungs 8 days (**E**) or 14 days (**F**) following intranasal infection of mice with 3 × 10^4^ yeasts. Individual data points are shown (*n* = 3–4 mice) with horizontal bars representing the mean fungal burden in the lungs. Asterisks indicate significant differences compared to the wild type by Student’s *t*-test (***P < 0.001).

The presence of a PTS1 sequence at the *C*-terminus of Sid1 and Sid3, and the requirement for peroxisomes in siderophore production, suggests the Sid1 and Sid3 proteins should localize to the peroxisome matrix. Bioinformatic surveys of fungal genomes for homologs of siderophore-producing enzymes found PTS1 sequences on *H. capsulatum* Sid3 and an *H. capsulatum* homolog of the *Aspergillus* SidH protein (24). To confirm this, we constructed an *N*-terminal RFP fusion to Sid1 and Sid3 to preserve the *C*-terminal PTS1 and monitored their localization by fluorescence microscopy. To validate the RFP:Sid1 fusion protein retained function, we first generated a knockout of the *SID1* gene using CRISPR-Cas9 (CRISPR-associated protein) methodology (50) and complemented the *sid1* deletion mutation with the expression of the RFP:Sid1 fusion protein. Expression of the RFP:Sid1 fusion protein in the *sid1* mutant restored the ability of yeasts to grow in limited iron media, confirming functional complementation (Fig. S5). The RFP:Sid1 protein localized to puncta similar to peroxisomes, and the peroxisomal localization was lost when the fusion protein was expressed in the Pex5-depleted strain that lacks PTS1-protein import (Fig. 5A). The peroxisomal localization of RFP:Sid1 is also lost in *pex10* and *pex33* mutant strains and can be restored by complementation (Fig. S6). Furthermore, the RFP:Sid1 protein co-localized with the GFP:PTS1-marked peroxisomes (Fig. 5B), demonstrating that Sid1 is transported to the peroxisome via the PTS1-import pathway. The *H. capsulatum* Sid3 fusion protein to RFP also localized in a punctate pattern and showed strong colocalization with GFP:PTS1-marked peroxisomes (Fig. 5B), linking two distinct proteins encoded in the siderophore biosynthetic gene cluster to peroxisomes.

### Siderophore production is required for *H. capsulatum* virulence only during adaptive immunity

Although both PTS1- and PTS2-protein peroxisomal import pathways are important for *H. capsulatum* siderophore production, the requirement for each differs during mammalian infection. To determine when siderophore production is necessary during host infection, we examined the lung fungal burden established by the Sid1-depleted strain, which is deficient for siderophore biosynthesis. Two previous studies of *H. capsulatum* Sid1 conflict as to when mammalian infection requires siderophore production (10, 51). In the current study, preventing siderophore biosynthesis (*SID1*-RNAi) attenuates *H. capsulatum* yeast growth in cultured macrophages (Fig. 5C) and decreased macrophage death (Fig. 5D). However, infection of mice with the Sid1-depleted strain produced an equivalent fungal burden in lungs as that of Sid1-expressing yeasts at 8 days post-infection (Fig. 5E) but was strongly attenuated at 14 days (Fig. 5F) similar to the results with the PTS2-protein import pathway-deficient strain (*PEX7*-RNAi; Fig. 3C and D).

### Peroxin Pex11 is required for *H. capsulatum* virulence in a siderophore-independent manner

**Fig 6:**
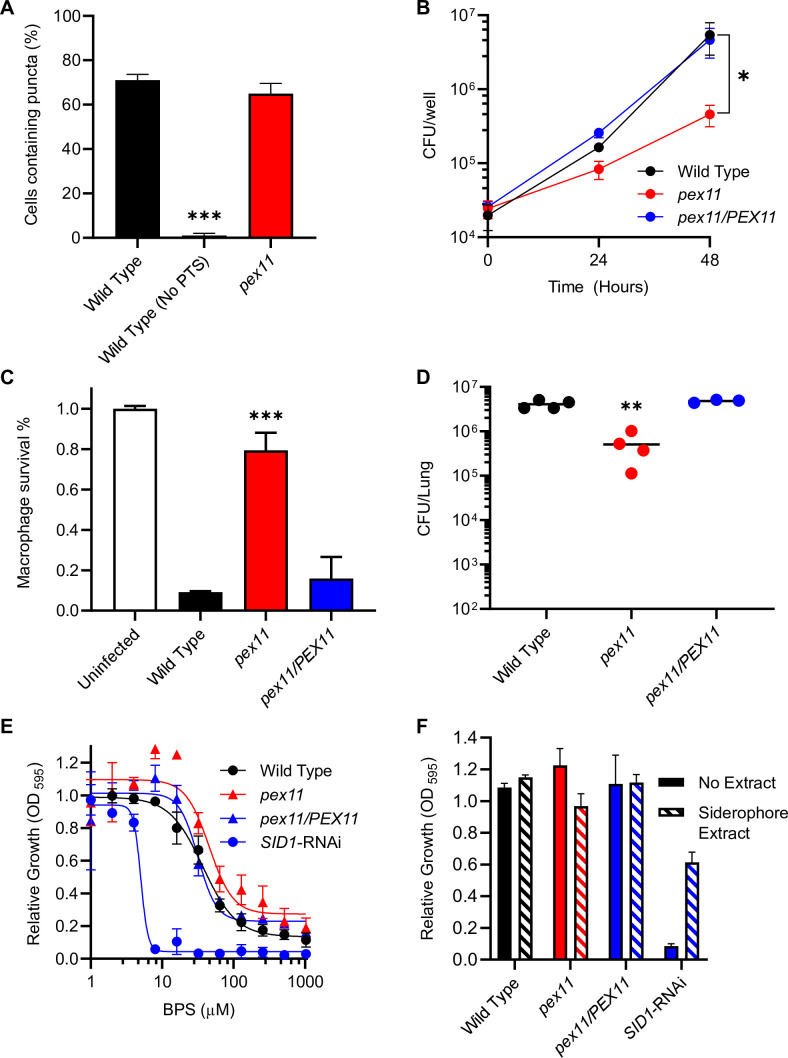
Pex11 is required for virulence in a siderophore- and PTS1-independent manner. The contribution of Pex11 to *H. capsulatum* peroxisome import (**A**), intracellular proliferation (**B**), virulence in cultured macrophages (**C**), virulence *in vivo* (**D**), and siderophore production (**E and F**) was determined using a Pex11-deficient mutant strain (*pex11;* red) and compared to wild-type (Wild Type; black) or Pex11-complemented (*pex11/PEX11;* blue) yeasts. (**A**) Peroxisome-localization of peroxisome-targeted GFP (GFP:PTS1) was quantified in wild-type and Pex11-deficient strains by determining the percentage of cells containing subcellular puncta. Wild-type cells expressing GFP lacking the PTS1 signal [Wild Type (No PTS)] served as the background control. Data represent the average (± SD) of biological replicates (*n* = 3) with 150 yeasts cells scored per replicate for each strain. (**B**) Proliferation of Pex11-deficient yeasts in macrophages was quantified by measurement of CFUs after serial dilution onto solid media. Data points represent the mean number of CFUs (± SD) from biological replicate infections (*n* = 3). (**C**) Survival of *H. capsulatum*-infected macrophages was quantified after 7 days of infection by measurement of the remaining macrophage-derived β-galactosidase activity. Data represent the mean surviving macrophages (± SD) from biological replicate infections (*n* = 3). (**D**) The virulence of Pex11-deficient yeasts *in vivo* was determined by quantifying the fungal burden (CFU) of *H. capsulatum* in lungs 8 days following intranasal infection of mice with 3 × 10^4^ yeasts. Individual data points are shown (*n* = 3–4 mice) with horizontal bars representing the mean fungal burden in the lungs. (**E**) Dose-response of *H. capsulatum* Pex11-deficient yeast growth in graded concentrations of the iron chelator BPS. Data points indicate the mean growth (± SD) of yeasts in BPS normalized to the wild-type growth in the absence of BPS. Curves represent a four-parameter non-linear regression fit to the data. (**F**) Rescue of Pex11-deficient growth in limited iron by exogenous siderophores was determined by growing yeasts in 20 µM BPS with 0.25% (vol/vol) water (No Extract; solid bars) or in 20 µM BPS supplemented with 0.25% (vol/vol) of a siderophore extract (Siderophore Extract; hatched bars). Growth of yeasts was normalized to the growth of each strain in standard HMM media (contains 3 µM FeSO_4_), and the data represent the average relative growth (± SD) of biological replicates (*n* = 3). Asterisks indicate significant differences compared to the wild type (*P < 0.05, **P < 0.01, ***P < 0.001).

In addition to *PEX5*, *PEX10*, and *PEX33*, the genetic screen identified another peroxin-encoding gene required for *H. capsulatum* intracellular growth. In this mutant (199G8), the T-DNA element was inserted into the third exon of the gene (879 nucleotides downstream of the CDS start) encoding the ortholog of the *S. cerevisiae* Pex11 peroxin. Unlike the *pex5*, *pex10*, and *pex33* mutants, loss of Pex11 function did not impair the import of a PTS1-tagged GFP into peroxisomes (Fig. 6A), distinguishing the role of Pex11 from that of the other peroxin mutants isolated. Nonetheless, loss of Pex11 function impaired the intracellular proliferation of yeasts (Fig. 6B) and decreased macrophage killing by yeasts (Fig. 6C), which was restored by complementation of the *pex11* mutation through the expression of the *PEX11* gene. Importantly, Pex11-deficient yeasts had attenuated virulence in respiratory infections at 8 days compared to the *PEX11*-expressing strain (Fig. 6D). However, the *pex11* mutant did not show decreased growth in iron-limited media or dependence on siderophore utilization (Fig. 6E and F), confirming this peroxin contributes to *H. capsulatum* pathogenesis through a mechanism independent of the siderophore biosynthesis role of peroxisomes.

## DISCUSSION

The residence of *H. capsulatum* yeasts within phagosomes imposes obstacles to the growth and replication of the yeasts. In this study, we establish that peroxisome functions are necessary for *H. capsulatum* intracellular proliferation and virulence. These pathogenesis-related functions do not include the canonical role of the peroxisome in fatty acid catabolism as depletion of the enzymes fatty acid oxidase (Fox1) and isocitrate lyase (Icl1) do not attenuate *H. capsulatum* virulence (9). *C. neoformans* yeasts are also internalized by phagocytes, but like the situation in *H. capsulatum*, there is no requirement for isocitrate lyase in *C. neoformans* intracellular growth (52). This contrasts with the demonstrated requirement of the glyoxylate shunt for *C. albicans* virulence (39), suggesting differing strategies for yeasts within phagocytes as well as differences in the degree to which *H. capsulatum* and *C. albicans* use the phagocyte as the host niche for infection.

The identification of multiple components of the peroxisome that attenuate *H. capsulatum* intracellular growth indicates a distinct role for peroxisomes in *H. capsulatum* pathogenesis defined by the factors that comprise the peroxisome matrix. Depletion or loss of the Pex5 and Pex7 cytosolic receptors for PTS1 and PTS2, respectively, attenuates *H. capsulatum* virulence, indicating a requirement for specific peroxisome matrix proteins at different stages of infection. A catalog of PTS1-containing proteins encoded in the *H. capsulatum* genome identified multiple components of siderophore biosynthesis. At least two siderophore biosynthetic enzymes, Sid1 and Sid3, localize to the peroxisome, and the peroxisomal localization of Sid1 is dependent on Pex5 (Fig. 5A and B). Depletion or disruption of multiple peroxisome import components, including the PTS1 and PTS2 pathways, impairs *H. capsulatum* acquisition of iron showing proper peroxisome function is necessary for *H. capsulatum* siderophore production (10, 53).

Related *H. capsulatum* strains 505 and G184 produce fusarinine and coprogen-type hydroxamate siderophores in culture (54). The *H. capsulatum* genome encodes many proteins homologous to the *Aspergillus fumigatus* siderophore biosynthetic pathway (24, 55) with most genes present in a single iron-regulated gene cluster (10), including the *SID1*, *SID3*, *SID4*, and *NPS1* genes. *SID1* encodes an N5-monooxygenase, which catalyzes the first committed step of siderophore biosynthesis by converting l-ornithine into N5-hydroxy-l-ornithine. Interestingly, the *H. capsulatum* Sid1 protein also has a PTS1 signal (ARL) and shows peroxisomal localization (Fig. 5A and B) unlike the orthologous *A. fumigatus* SidA protein that lacks a *C*-terminal PTS1 sequence (24, 55). Sid1 orthologs from related dimorphic fungi, such as *Blastomyces, Paracoccidioides,* and *Coccidioides* contain putative PTS1 sequences as well. Close inspection of the Sid1:RFP localization shows that some Sid1 is also present in the cytosol in contrast to the Sid3:RFP (Fig. 5B). This might reflect different efficiency of PTS1 recognition and capture of the Sid3 and Sid1 PTS1 (SKL and ARL, respectively). Compartmentalization of the Sid1 N5-hydroxy-l-ornithine monooxygenase within the peroxisome in Onygenales fungi with other siderophore biosynthetic enzymes (e.g., Sid3) may concentrate enzyme activity, substrates, and products for more efficient siderophore synthesis. Although *A. fumigatus* encodes proteins for both reductive iron transport and production of hydroxamate siderophores, only the latter is required for virulence *in vivo* (48, 56). The genome of the G217B strain of *H. capsulatum* used in this study does not encode a reductive iron acquisition system and thus relies on siderophores for the acquisition of limited iron (51). Together these findings indicate that fungal acquisition of iron from the mammalian host specifically requires the peroxisome-dependent biosynthesis of siderophores.

Depletion of the PTS1 and PTS2 pathways revealed distinct phenotypes for virulence *in vivo*, suggesting multiple roles for *H. capsulatum* peroxisomes in this context. Loss of the PTS1 pathway attenuates virulence early during infection, while loss of the PTS2 pathway only attenuates virulence at late stages of infection in a murine model. Sid4, encoded in the *H. capsulatum* siderophore gene cluster (10), contains a putative PTS2 localization sequence (RLQQTLNHI) as previously identified (24), linking the PTS2 import pathway and siderophore production in *H. capsulatum* (24). This, in combination with the late-stage attenuation defect seen in strains that lack Sid1 or Pex7, suggests that the virulence attenuation of *H. capsulatum* yeasts lacking the PTS2 protein import pathway can be explained primarily by the loss of siderophore production. The dynamic requirement for fungal siderophores during infection suggests that iron is available for *H. capsulatum* in the phagosome during the early stages of infection, but becomes limiting at later time points, necessitating the use of siderophores to acquire nutritional iron (10). This finding echoes a previous study that showed phagosomal copper availability to *H. capsulatum* becomes scarce only during adaptive immunity (4). This constitutes a trend of metal sequestration (both iron and copper) from intracellular *H. capsulatum* via adaptive immune responses as a form of nutritional immunity designed to limit the proliferation of intracellular pathogens.

While both PTS1- and PTS2-protein import into peroxisomes is required for siderophore production and iron acquisition during infection, the early stage virulence attenuation resulting from the loss of PTS1-protein import indicates another role for peroxisomes in *H. capsulatum* pathogenesis distinct from siderophore biosynthesis. The difficulty in plating of PTS1-protein import-deficient strains but not PTS2-protein import-deficient strains further underscores the separable roles of peroxisomes in *H. capsulatum* biology. Synthesis of the vitamin biotin in *A. fumigatus* relies on the PTS1-protein import pathway in that organism, suggesting some essential vitamin synthesis requires peroxisomes (21). However, previous studies have determined that the loss of biotin biosynthesis does not significantly attenuate *H. capsulatum* growth in macrophages and virulence *in vivo* (57), which suggests biotin deficiency is not the cause of the peroxin mutants’ attenuation. Peroxisomes are notable for sequestering peroxide-generating enzymatic reactions and consequently harbor a peroxisomal catalase. In *H. capsulatum*, the peroxisomal catalase, CatP, contains a PTS1 sequence which is sufficient to direct peroxisomal import (Fig. 2A). However, depletion of the peroxisomal catalase CatP does not impair *H. capsulatum* virulence during infection due to a redundant cell-surface-localized catalase, CatB (42), indicating the loss of peroxisomal CatP localization does not underlie the strong virulence attenuation of the peroxisomal mutants. These data suggest a novel role for peroxisomes during mammalian fungal infection.

The observation that the Pex11 protein is required for full virulence further indicates peroxisomal functions independent of siderophore biosynthesis are required for *H. capsulatum* pathogenesis and may give insight into the role of this organelle. Pex11 is central to the peroxisomal division process (58) and defects in Pex11 in both mammalian cells and yeasts generally result in a reduction of peroxisome abundance (59 - 61). However, the loss of Pex11 function in *H. capsulatum* did not eliminate peroxisomes (Fig. 6A) and did not prevent siderophore biosynthesis (Fig. 6E and F). Nonetheless, the absence of Pex11 function attenuated *H. capsulatum* virulence similar to the loss of PTS1-protein import machinery, indicating a distinct role from the function of peroxisomes in siderophore production. In addition, pex11 mutants do not have defects in plating efficiency, unlike the PTS1-protein import mutants. Thus, one or more specific PTS1-pathway imported matrix protein functions are necessary for *H. capsulatum* virulence with some linked to Pex11 function. This Pex11-dependent and PTS1-protein import-dependent function remains undetermined, but a report has suggested that Pex11 could be a non-selective pore-forming protein that transfers metabolites smaller than 400 Da across the peroxisomal membrane (62). In light of this, we hypothesize that Pex11 may provide substrates for a PTS1-containing matrix protein that enables *H. capsulatum* virulence during the early stages of infection.

This study demonstrates the multifaceted role of peroxisomes in the growth and replication of an intracellular fungal pathogen. The *H. capsulatum* peroxisome has at least three functions that are separable based on the distinct phenotypes of the isolated peroxin mutants: (i) siderophore production, which is essential for virulence during the adaptive immune response, (ii) an undefined mechanism contributing to plating efficiency, and (iii) a novel virulence function essential for early stage infections that may involve transport of small molecules as a substrate for a peroxisomal enzyme. Future studies focused on PTS1-containing peroxisome matrix proteins should uncover the different functions of the peroxisome and how they contribute to intracellular proliferation and virulence of *H. capsulatum*. Given the importance of fungal peroxisomes to multiple aspects of fungal virulence, including pathogenesis-enabling siderophore production, the peroxisome machinery could represent a potential target for antifungal therapeutics, perhaps by targeting the Pex33 peroxin, which appears specific for ascomycetous fungi.

## MATERIALS AND METHODS

### *H. capsulatum* strains and growth

The *H. capsulatum* strains used in this study (Table S2) are derived from the North American clade 2 clinical isolate G217B (ATCC 26032). *H. capsulatum* yeasts were grown in *H. capsulatum*-macrophage medium (HMM) (63) with continuous shaking (200 rpm) at 37°C. Yeasts were grown to the exponential phase for use in growth and infection studies. Cell density was quantified by measurement of culture turbidity (optical density at 595 nm). For growth on the solid medium, HMM was solidified with 0.6% agarose and supplemented with 25 µM FeSO_4_. For dose–response tests with the chelator BPS, strains were grown in HMM at 37°C in microtiter plates with continuous shaking, and cell density was assessed at 72 hours.

### Macrophage cell culture

The lacZ-expressing P388D1 cell line was created from the murine macrophage cell line P388D1 [ATCC CCL-46 (64)]. LacZ-expressing P388D1 macrophage cells were maintained in Ham’s F-12 medium supplemented with 10% fetal bovine serum (FBS; Atlanta Biologicals) and cultured at 37°C in 5% CO_2_/95% air. For infection experiments, yeasts were added to macrophages [multiplicity of infection (MOI) of 1:1], and surviving macrophages were quantified by determining the remaining β-galactosidase activity (64).

### Isolation of *H. capsulatum* mutants with attenuated intramacrophage growth

*H. capsulatum* strain WU15 or strain OSU233 (63) was used as the genetic background for insertional mutagenesis using *Agrobacterium tumefaciens* strain LBA1100 harboring plasmid pBHt2 (9). Individual mutants were used to infect monolayers of P388D1 lacZ-expressing macrophage cells (64). Mutants with at least a 30% reduction in intramacrophage yeast growth (red fluorescence) or at least a 30% reduction in lysis of the macrophages were retained as intracellular-proliferation-deficient mutants.

### Mapping and complementation of T-DNA insertional mutants

The location of the T-DNA insertion in individual mutants was determined by thermal asymmetric interlaced PCR (TAIL-PCR) as described previously (4). The sequences were aligned to the *H. capsulatum* genome sequence to determine the genomic context of the T-DNA insertions. The mutants characterized in this study included 79C10 (OSU197), 07A9 (OSU9), 502F2 (OSU131), and 199G8 (OSU377).

To complement the *pex10* and *pex33* mutants, a fragment consisting of the corresponding gene sequence was amplified by PCR from *H. capsulatum* G217B genomic DNA using gene-specific primers and cloned into a *URA5*-based T-DNA plasmid (pCR628) for constitutive expression from the *H. capsulatum H2B* promoter. To complement the *pex11* mutant, a 1,841-bp fragment consisting of the *PEX11* gene sequence and 194 bp upstream and 562 bp downstream was amplified by PCR from *H. capsulatum* G217B genomic DNA using gene-specific primers. Complementation vectors or a control green fluorescent protein (GFP) expression vector (pCR628) were transformed by *A. tumefaciens*-mediated transformation into the corresponding peroxin mutants.

### Depletion of peroxin and siderophore biosynthesis gene functions by RNAi

Peroxin and siderophore biosynthesis gene functions were depleted from *H. capsulatum* yeasts by RNA interference (RNAi) (65). The RNAi vector was created by the insertion of two copies of a region of the targeted gene coding region [coding DNA sequence (CDS)] in an inverted orientation into the RNAi sentinel vector pED02 (66). RNAi vectors were transformed by *Agrobacterium*-mediated transformation into the GFP-expressing uracil auxotroph sentinel strain OSU194. Uracil prototroph (Ura+) transformants were recovered, and the sentinel GFP fluorescence was quantified using a modified gel documentation system and ImageJ software (v1.44p; http://imagej.nih.gov.proxy.lib.ohio-state.edu/ij) to identify transformants with depletion of target gene expression.

### Intramacrophage proliferation of *H. capsulatum* yeasts

Macrophage monolayers were established in 96-well plates and were infected with yeasts at an MOI of 1:1. After 2 hours, the medium was replaced to remove the remaining extracellular yeasts. Immediately or at 24 or 48 hours post-infection, intracellular yeasts were quantified by removal of macrophage culture supernatant followed by lysis of the macrophages with sterile H_2_O and plating of serial dilutions of the macrophage lysate on solid HMM to enumerate CFUs. For *H. capsulatum* strains unable to form CFUs, yeasts were instead enumerated directly by counting using a hemacytometer. Hoechst 33342 dye (1 µg/mL) was added to the macrophage lysate before counting, and unstained yeasts were deemed viable.

### Murine model of pulmonary histoplasmosis

Wild-type C57BL/6 mice were infected with wild-type, mutant, or complemented *H. capsulatum* strains by intranasal delivery of approximately 3 × 10^4^ yeast cells. At 8 or 14 days post-infection, mice were euthanized and their lungs were collected for analysis. For CFU enumeration, serial dilutions of lung homogenates were plated on solid HMM to determine the fungal burden. For qPCR enumeration of fungal genomes, lungs were homogenized in water and passaged 20 times through a 25-gauge needle to lyse remaining mammalian cells. Yeasts were collected by centrifugation (16,000× *g* for 2 minutes), and the yeast-containing pellet was resuspended in 200 µL water. DNA was extracted by mechanical disruption of yeasts with 0.5-mm-diameter glass beads followed by isopropanol precipitation of DNA. *H. capsulatum* DNA was quantified by probe-based qPCR using a custom TaqMan probe (TTCTAGACGCTCTCAAGGGCGTTCTCAAG with 5′ 6-FAM (6-carboxyfluorescein) label and ZEN/Iowa Black dual quencher; Integrated DNA Technologies) targeting the *H. capsulatum RPS12* gene. Forward and reverse *RPS12* primers with sequences TCGGACGGAGAGACCGCT and TCACGGAGGACAACGCAAGAGC, respectively, were used for qPCR with the *RPS12* TaqMan probe, with Entrans 2X qPCR Master Mix (Abclonal). For histology analysis, lungs were fixed in 10% formalin, paraffin-embedded, and sectioned. Grocott’s methenamine silver stain was performed on three non-consecutive lung sections from each mouse to allow the identification of *H. capsulatum* in lung tissue. A semi-quantitative measure of *H. capsulatum* fungal burden was obtained by counting yeast from 20 different non-overlapping 40× image fields from each lung section (three sections per lung). For CFU enumeration, serial dilutions of the homogenates were plated on solid HMM to determine the fungal burden (CFU). Animal experiments were performed in compliance with the National Research Council’s Guide for the Care and Use of Laboratory Animals and were approved by the Institutional Animal Care and Use Committee (IACUC) at Ohio State University (protocol 2007A0241).

### Localization of Sid1 and Sid3 prot9eins

The wild-type *H. capsulatum SID1* gene and *SID3* genes were amplified by PCR and cloned into a T-DNA plasmid (pAG22) fusing the coding sequences to sequence to an *N*-terminal td-Tomato red-fluorescence protein (RFP) gene. The resultant plasmids (pCS22 and pCS12) were transformed into *H. capsulatum* yeasts by *A. tumefaciens*-mediated transformation. The RFP:Sid1 expression construct was used to complement a *sid1* deletion mutation.

Directed mutation of the *SID1* locus was done by CRISPR/Cas9-mediated gene editing using a Cas9 and CRISPR guide RNA (gRNA) expression plasmid [derived from pPTS608 (67)] and optimized methodology (50). The gRNA protospacer sequences GAACGCGACGCTGAATCCCG and CGAAGAGTTGACATACACCA were used to target the *H. capsulatum SID1* locus.

### Extraction of siderophores from conditioned *H. capsulatum* media

*H. capsulatum* strain WU15 was grown in 250 mL of 3M media (63) with 2% glucose, 30 mM glutamate, 100 µg/mL uracil, and with either 5 µM FeSO_4_ (siderophore-repressing condition) or no supplemented FeSO_4_ (siderophore-inducing condition). After 10 days of growth, culture filtrate was recovered by removal of yeasts by centrifugation (16,000× *g* for 5 minutes) and filtration of the supernatant (0.2-µm-diameter pore membrane). Diaion HP20 synthetic adsorbent resin (Alfa Aesar) was added to each sample (1 g/50 mL) and mixed at 30°C for 1 hour. The resin was collected, transferred to a gravity column, and washed with 10 mL of ultrapure H_2_O. Elution fractions were then obtained by flowing 2 mL of increasing aqueous methanol (10% to 100%) through the column. Fractions were evaporated at 60°C overnight, and the material was resuspended in 1 mL of H_2_O. Siderophore-containing fractions were identified via a modified Chrome Azurol S assay (68). Briefly, 0.1 mL of each fraction was mixed with 0.3 mL of water and 0.4 mL of CAS assay solution (600 µM hexadecyltrimethyl ammonium, 15 µM FeCl_3_, 150 µM Chrome Azurol S, 200 mM MES [2-(N-morpholino) ethanesulfonic acid], 0.5 mM HCl, and 5 mM 5-sulfosalicylic acid) and incubated for 1 hour. Siderophore scavenging of iron from the iron-containing CAS complex was determined as the absorbance ratio (420 nm:630 nm) measured with a spectrophotometer. For siderophore rescue assays, the extract originating from the 50% methanol fraction was added to HMM media with 20-µM BPS to a final concentration of 0.25% (vol/vol). Corresponding fractions from cultures grown in siderophore synthesis-repressing conditions (i.e., fractions from cultures supplemented with 5 µM FeSO_4_) were used as the control. The growth of *H. capsulatum* yeasts in siderophore-lacking and siderophore-supplemented media was measured by optical density (595 nm) after 72 hours and normalized to the growth of each strain in HMM with no BPS and no siderophores were added.

## SUPPLEMENTAL MATERIAL

Supplemental Material mbio.03284-22-s0001.pdf Supplemental Figures and Tables.

ASM does not own the copyrights to Supplemental Material that may be linked to, or accessed through, an article. The authors have granted ASM a non-exclusive, world-wide license to publish the Supplemental Material files. Please contact the corresponding author directly for reuse.
