# Supplementary material for: Histoplasma capsulatum requires peroxisomes for multiple virulence functions including siderophore biosynthesis
Source: mBio. 2023 Jul 11;14(4):e03284-22. doi: 10.1128/mbio.03284-22 (PMC10470777; doi:10.1128/mbio.03284-22)
Supplement: Supplemental Material — Supplemental Figures and Tables. [file mbio.03284-22-s0001.pdf]

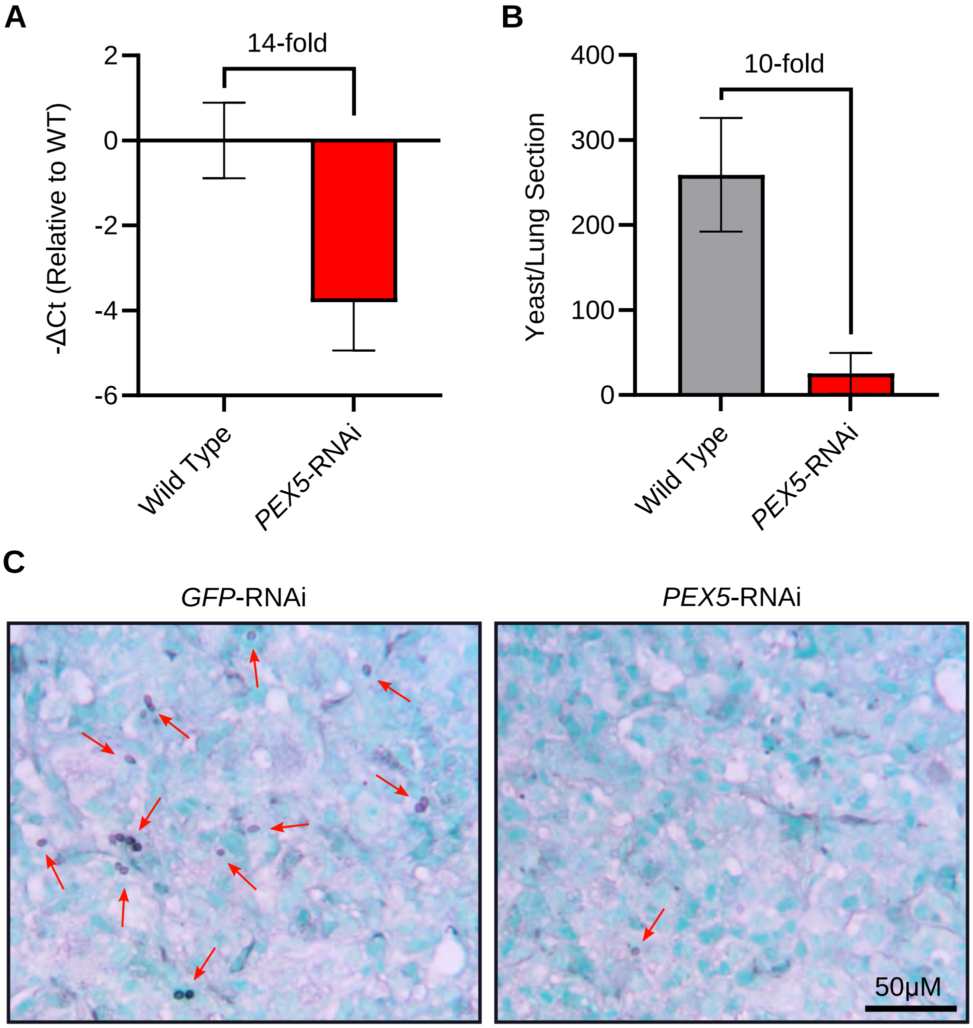

Figure S1: Depletion of PEX5 reduces fungal burden *in vivo*. The virulence of Pex5-deficient yeasts (*PEX5*-RNAi) *in vivo* was determined by measuring the number of fungal genomes in lung tissue by quantitative-PCR (A) or by quantifying the number of yeast cells in lung tissue by histology (B-C). (A) Lungs from Pex5-deficient yeasts (*PEX5*-RNAi) or Pex5-expressing yeasts (*GFP*-RNAi) used for intranasal infection of mice were homogenized and total DNA extracted. Fungal genomes were quantified via TaqMan probe-based qPCR specific for the *H. capsulatum RPS12* gene (probe=TTCTAGACGCTCTCAAGGGCGTTCTCAAG). Fold change was calculated using ΔCt values (*GFP*-RNAi vs *PEX5*-RNAi infected lungs), with each cycle difference in ± amplification representing a two-fold decrease in the number of genomes present. Data represents the mean *H. capsulatum* genome equivalents (± standard deviation) relative to *GFP*-RNAi from biological replicate infections (n=3 mice). (B) *H. capsulatum* burden was quantified by microscopy in GMS-stained lung sections. Data represent the mean total count of yeasts (± standard deviation) from 60 fields per lung from biological replicate infections (n=3). (C) Representative images are shown of GMS staining of lung sections after eight days of infection with *GFP*-RNAi and *PEX5*-RNAi strains of *H. capsulatum*. Red arrows denote *H. capsulatum* yeast cells defined by both GMS staining and morphology.

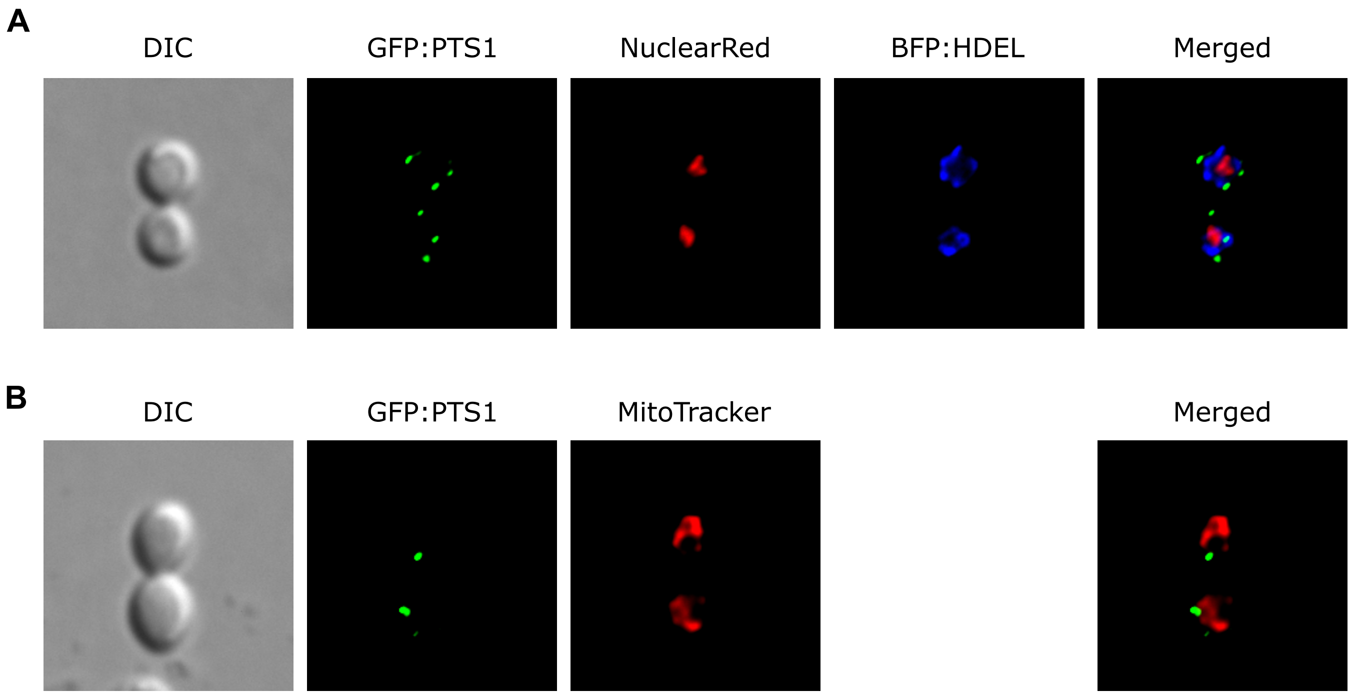

Figure S2. Peroxisome-targeted GFP does not colocalize with nuclei, mitochondria, or endoplasmic reticulum. *H. capsulatum* yeast cells were collected from liquid culture and the peroxisomal-targeted GFP was observed using fluorescence microscopy for colocalization with nuclear and ER makers (A) or mitochondria (B). Green-fluorescent protein was targeted to the peroxisome (GFP:PTS1) by inclusion of the C-terminal 3 amino acids representing the PTS1 signal from the peroxisomal catalase, CatP. Subcellular organelles were imaged by fluorescence by staining nuclei (Nuclear-ID Red, Enzo), mitochondria (MitoTracker Red CMX, Thermo), or by expression of a signal peptide-containing mTag blue-fluorescent protein (BFP) with a C-terminal HDEL sequence for retention in the endoplasmic reticulum (ER). Differential-interference contrast (DIC) images were obtained at 150X magnification and the corresponding fluorescence images deconvolved and single focal-plane images presented to indicate organelle subcellular localizations.

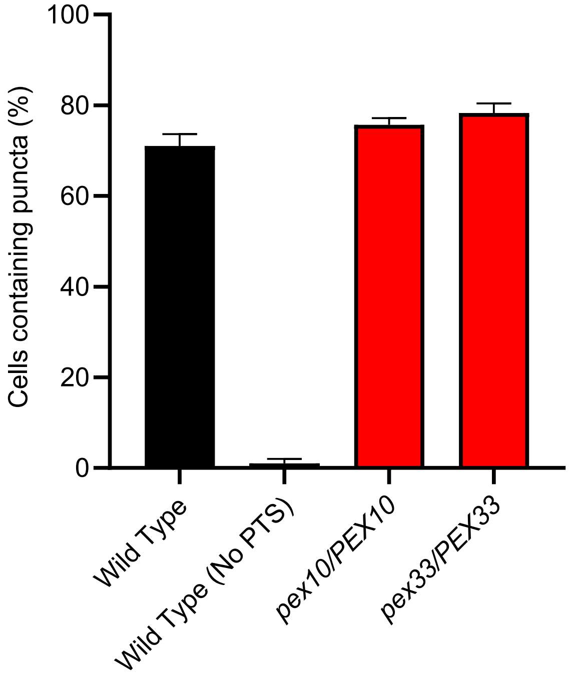

Figure S3: PTS1-dependent import of peroxisome-targeted proteins is restored by complementation of *pex10* and *pex33* mutations. Localization of peroxisome-targeted GFP was determined by microscopy for wild-type *H. capsulatum* yeasts and yeast with complemented *pex10* and and *pex33* genes. Peroxisomal localization of peroxisome-targeted GFP was determined by quantifying the localization of the GFP into subcellular puncta in wild-type and peroxin-complemented strains. The peroxisome-targeted GFP (GFP:PTS1) was expressed in wild-type cells (Wild Type) or peroxin-complemented yeast and the number of yeast cells in the population with GFP fluorescence localized to one or more subcellular puncta were quantified. Wild-type cells expressing GFP lacking the PTS1 signal (Wild Type (No PTS)) served as the background control. Data represent the average (± standard deviation) of biological replicates (n=3) with 150 yeasts cells scored per replicate for each strain.

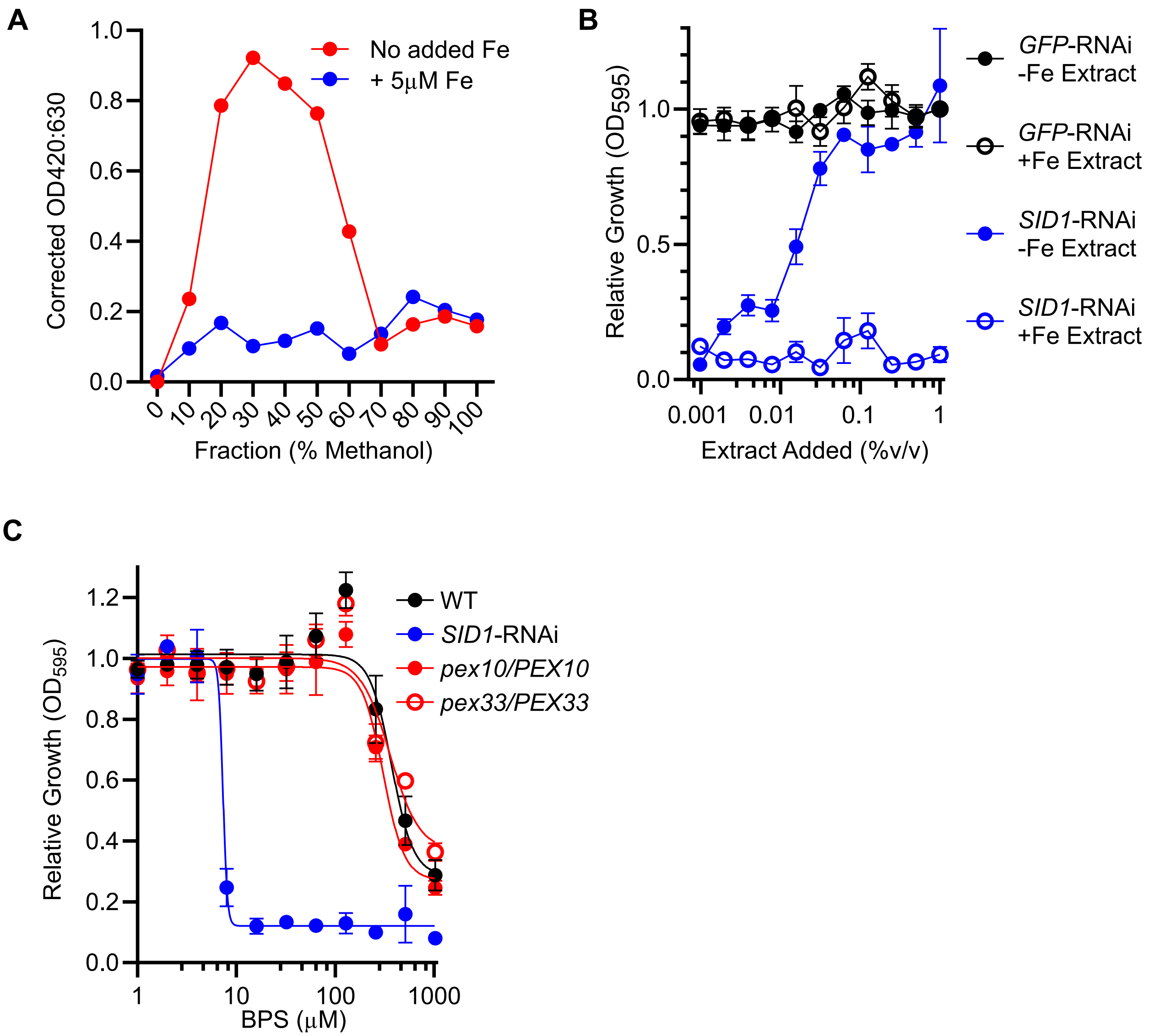

Figure S4: Rescue of siderophore-deficiency by siderophore-containing extract or complementation of pex10 and pex33 mutations. Extracts containing siderophores were prepared from wild-type *H. capsulatum* yeast culture filtrates (A) and added to yeasts unable to produce siderophores (*SID1*-RNAi) to test for rescue of growth (B). (A) *H. capsulatum* culture filtrate prepared from yeasts cultured in media lacking added iron (no added Fe; red) or from yeasts cultured with 5µM iron (+ 5µM Fe; blue) was extracted using Diaion resin and fractions from increasing methanol concentration elutions tested for siderophore activity using a chrome azurol S iron release assay. 50% methanol was determined to be sufficient to elute the majority of siderophores from the Diaion resin and was used for supplementation of media. B) Restoration of the growth (optical density at 595nm) of yeasts unable to produce siderophores (*SID1*-RNAi) by dose-dependent supplementation of the siderophore-containing extract from the 50% elution fraction. Siderophore-producing yeasts (*GFP*-RNAi; black) or yeasts deficient in siderophore production (*SID1*-RNAi; blue) were cultured in the presence of 50% methanol fractions prepared from yeasts grown in the absence of added iron to stimulate siderophore production (-Fe extract; solid symbols) or supplemented with 5µM $FeSO_4$ to repress siderophore biosynthesis (+Fe Extract; open symbols). (C) Dose response of *H. capsulatum* yeast growth in graded concentrations of the iron chelator BPS was tested for *PEX10* and *PEX33* complemented *pex10* and *pex33* mutants (red) and compared to growth of siderophore-deficient yeasts (*SID1*-RNAi; blue). Data points were normalized to the growth of wild-type in the absence of BPS. Data points represent the mean growth (± standard deviation) of biological replicates (n=3) and curves represent curve fit by four-parameter non-linear regression.

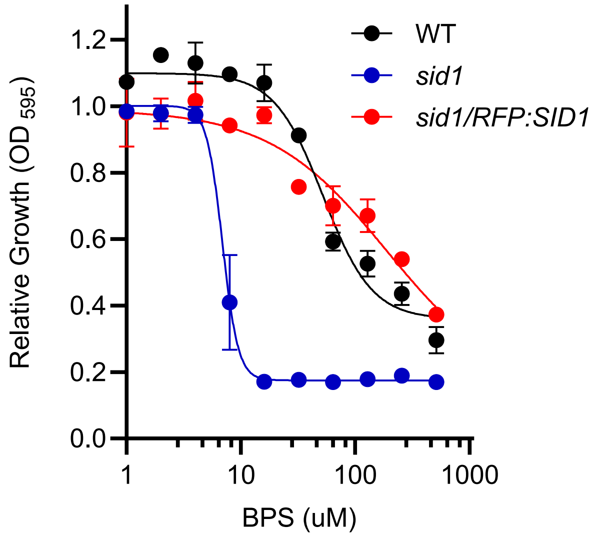

Figure S5: Expression of the RFP:Sid1 fusion protein functionally complements loss of Sid1 in *H. capsulatum*. Dose response of *H. capsulatum* yeast growth in graded concentrations of the iron chelator BPS were determined for wild-type yeasts (WT; black), a *sid1* deletion mutant (*sid1*, blue) and the *sid1* mutant expressing a RFP:Sid1 fusion protein (*sid1*/*RFP:SID1*). Data points were normalized to the growth of wild-type in the absence of BPS. Data points represent the mean growth (± standard deviation) of biological replicates (n=3) and curves represent curve fit by four-parameter non-linear regression.

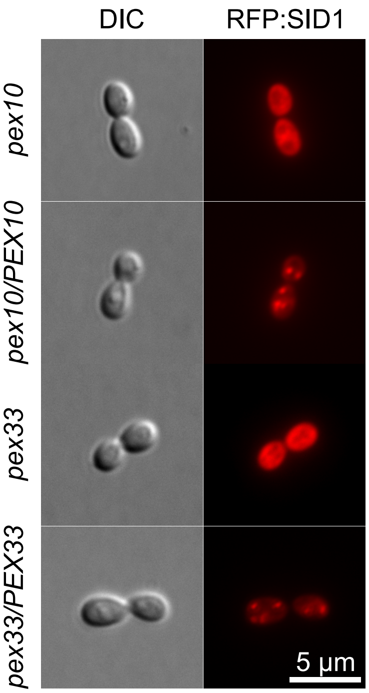

Figure S6: Localization of Sid1 to peroxisomes in *H. capsulatum* is dependent on Pex10 and Pex33. Differential interference contrast (DIC) and fluorescence microscopy images show the localization of an N-terminal translational fusion of RFP to Sid1 (RFP:Sid1) in pex10 and pex33 mutants and corresponding complemented strains. Scale bar represents 5µm.

Table S1: PTS1-containing proteins in *H. capsulatum*

Protein	C Terminal Sequence	Annotated *Histoplasma* Protein[1]	Top BLAST Hit other fungi[2]	Annotation/Putative Function
00150	SKL	KAG5301259.1	XP_755971.1	thioesterase family protein
00215	AKL	KAG5301183.1	XP_755349.1	phenylacetyl-CoA ligase
00338	SRL	KAG5300387.1	XP_746407.1	conserved hypothetical protein
00339	PRL	KAG5300388.1	XP_753079.1	long chain fatty alcohol oxidase
00411	AKL	KAG5300466.1	XP_749938.1	enoyl-CoA hydratase/ isomerase family protein
00458	AKL	No Hits	XP_752940.1	phosphoglycerate mutase
00481	SKL	KAG5300536.1	XP_752822.1	general amidase
00509	AKL	KAG5300564.1	XP_752810.1	hydroxyisocaproate dehydrogenase
00600	SRL	KAG5291931.1	XP_050469089.1	isocitrate dehydrogenase
00623	ARL	KAG5291953.1	XP_748293.1	FAD dependent oxidoreductase
00639	PRL	KAG5291971.1	No Hits	N/A
00703	AKL	KAG5292058.1	XP_753520.1	D-amino acid oxidase
00732	SKL	KAG5292095.1	XP_657810.1	Acyltransferase
01285	AKL	KAG5289247.1	XP_755585.1	carnitine acetyl transferase
01580	PKL	KAG5291489.1	XP_748141.1	a-pheromone processing metallopeptidase Ste23
01585	AKL	KAG5291497.1	XP_748136.1	dephospho-CoA kinase
01596	PKL	KAG5291508.1	XP_680900.1	phenylacetyl-CoA ligase
01648	AKL	No Hits	No Hits	N/A
01710	SKL	KAG5298195.1	XP_748661.1	mevalonyl-CoA hydratase
01778	AKL	KAG5298074.1	XP_755469.1	bifunctional fatty acid transporter/acyl-CoA synthetase
02031	SRL	KAG5298366.1	XP_050468705.1	glucose-6-phosphate 1-epimerase
02082	SKL	KAG5298315.1	XP_664356.1	acyl-CoA oxidase aoxA
02093	AKL	KAG5298303.1	XP_751599.1	toxin biosynthesis protein
02245	SKL	KAG5295228.1	XP_754167.1	copper amine oxidase
02368	SKL	KAG5292547.1	XP_747283.1	acetoacetyl-CoA synthase
02411	SKL	KAG5301693.1	XP_754855.1	enoyl-CoA hydratase/isomerase
02452	PKL	KAG5292411.1	XP_011392920.1	urea transporter
02741	ARL	KAG5290233.1	XP_011393182.1	fumarylacetoacetate hydrolase
02937	ARL	KAG5289565.1	XP_750038.1	Phosphotransferase enzyme
03101	SRL	No Hits	No Hits	N/A
03162	SKL	KAG5295425.1	XP_660975.1	hypothetical protein
03234	SKL	KAG5295360.1	No Hits	N/A

03376	AKL	No Hits	XP_747282.1	ureidoglycolate hydrolase
03409	AKL	KAG5298464.1	XP_001481734.1	DSBA family oxidoreductase
03410	SRL	KAG5301674.1	XP_753030.1	short chain dehydrogenase
03431	AKL	KAG5301012.1	XP_747326.1	2-hydroxyphytanoyl-CoA lyase
03469	ARL	KAG5301046.1	No Hits	N/A
03506	SKL	No Hits	No Hits	N/A
03589	SKL	KAG5294522.1	XP_755660.1	alcohol dehydrogenase
04029	AKL	KAG5288071.1	XP_747916.1	medium-chain fatty acid-CoA ligase faaB
04345	SKL	KAG5293032.1	XP_746948.1	lipase/thioesterase family protein
04353	ARL	KAG5293025.1	XP_752658.2	epoxide hydrolase
04595	SHL	KAG5297868.1	XP_749702.2	siderochrome-iron transporter MirC
04611	PKL	KAG5297849.1	XP_755226.1	GDSL Lipase/Acylhydrolase family protein
04661	SRL	KAG5297796.1	XP_755175.1	peroxisomal membrane protein PEX17
04681	AKL	KAG5297774.1	XP_755161.1	NADP-dependent malic enzyme MaeA
04722	AKL	KAG5295008.1	XP_755113.1	acyl-CoA synthetase
04729	AKL	KAG5298417.1	XP_755107.1	cytochrome b5
04805	AKL	KAG5301098.1	XP_753230.2	AMP-binding enzyme
04816	SKL	KAG5301108.1	XP_753935.1	O-methyltransferase
04960	SKL	KAG5296605.1	XP_663838.1	protein sidF
04963	ARL	KAG5296603.1	XP_663427.1	L-ornithine N5-oxygenase SidA
05112	SRL	KAG5296795.1	XP_754607.1	fructosyl amino acid oxidasesarcosine oxidase
05132	SKL	KAG5296775.1	XP_748275.1	peroxisomal dehydratase
05295	ARL	KAG5302300.1	XP_751233.1	cytochrome c peroxidase
05394	AKL	KAG5302093.1	XP_956821.1	enoyl-CoA hydratase/isomerase
05433	SRL	No Hits	No Hits	N/A
05559	AKL	KAG5300614.1	XP_746469.1	aldehyde dehydrogenase
05685	SRL	KAG5300751.1	XP_664504.1	triosephosphate isomerase
05795	SKL	KAG5300861.1	XP_657697.1	hypothetical protein
05832	SRL	KAG5300903.1	XP_753475.1	LON domain serine protease
05934	SRL	KAG5291835.1	XP_753754.2	Woronin body protein HexA
05967	SRL	KAG5291797.1	XP_753728.1	DEAD box helicase
06031	AKL	KAG5291726.1	XP_753679.1	2-nitropropane dioxygenase family oxidoreductase
06058	SKL	KAG5296768.1	XP_747970.1	isopenicillin N-CoA epimerase
06098	CHL	KAG5298285.1	No Hits	N/A

06490	SRL	KAG5301875.1	XP_746498.1	mitochondrial cytochrome b2
06655	SRL	KAG5287972.1	XP_753622.2	salicylate hydroxylase
06753	AKL	KAG5301816.1	XP_755318.1	oxidoreductase, 2-nitropropane dioxygenase
06795	ARL	KAG5301772.1	XP_747216.1	choline oxidase
06863	PKL	KAG5301709.1	XP_868852.1	factor-independent urate hydroxylase uaZ
06870	PRL	KAG5301700.1	XP_663522.1	catalase catC
06896	SKL	KAG5301674.1	XP_746577.1	peroxisomal multifunctional betaoxidation protein
06967	AKL	KAG5301610.1	XP_658469.1	methyltransferase
07194	AKL	KAG5289834.1	XP_753642.2	acyl-CoA dehydrogenase
07298	SKL	KAG5289945.1	XP_746692.1	alcohol dehydrogenase
07311	PKL	KAG5289959.1	XP_958767.1	platelet-activating factor acetylhydrolase
07446	SKL	KAG5290098.1	XP_752031.1	oxidoreductase, 2-nitropropane dioxygenase family
07625	PRL	KAG5294604.1	XP_050469211.1	endo-1,3(4)-beta-glucanase
07712	PKL	No Hits	No Hits	N/A
07717	ARL	KAG5301421.1	XP_749113.1	conserved hypothetical protein
07952	AKL	KAG5297106.1	XP_751204.1	lipid transfer protein
08165	AKL	KAG5296998.1	XP_747723.1	malate synthase AcuE
08266	AKL	KAG5297531.1	XP_752124.1	mitochondrial cytochrome b2-like
08344	AKL	KAG5297451.1	XP_750435.1	protein phophatase 2C family
08678	SRL	KAG5287187.1	No Hits	N/A
08747	AKL	KAG5287256.1	No Hits	N/A

[1]Annotations were assigned by BLAST hits to publicly available *Histoplasma* annotated genome (BioProject PRJNA682643)

[2]Annotations were assigned by BLAST hits to *Aspergillus fumigatus* AF293, A*spergillus nidulans* FGSC A4, *Saccharomyces cerevisiae* S288c, *Schizosaccharomyces pombe* 972h, and *Neurospora crassa* OR74A.

Table S2: *H. capsulatum* strains[1,2]

WU15	*ura5-42Δ*
OSU9	*ura5-42Δ pex10-1*::pBHt2(hph)
OSU131	*ura5-42Δ pex33-1::pBHt2(hph)*
OSU194	*ura5-42Δ zzz::pAG21 (*$G418^R$*,GFP)*
OSU197	*ura5-42Δ zzz::pQS01 (*$G418^R$*, RFP) pex5-1::ppBHt2(hph)*
OSU233	*ura5-42Δ zzz::pQS01 (*$G418^R$*,* P_{TEF1}*-tdTomato RFP)*
OSU237	*ura5-42Δ pex10-1::pBHt2(hph) zzz::pCR628 (URA5,* P_{H2B}*-gfp)*
OSU238	*ura5-42Δ pex10-1::pBHt2(hph) zzz::pCR650 (URA5,* P_{H2B}*-PEX10)*
OSU239	*ura5-42Δ pex33-1::pBHt2(hph) zzz::pCR623 (URA5,* P_{TEF1}*-gfp)*
OSU240	*ura5-42Δ pex33-1::pBHt2(hph) zzz::pCR644 (URA5,* P_{H2B}*-PEX33)*
OSU277	*ura5-42Δ □□zzz::pCR639 (URA5,* P_{TEF1}*-gfp)*
OSU278	*ura5-42Δ □□zzz::pMG02 (URA5,* P_{TEF1}*-gfp:pts1)*
OSU279	*ura5-42Δ pex10-1*::pBHt2(hph) *zzz::pCR639 (URA5,* P_{TEF1}*-gfp)*
OSU280	*ura5-42Δ pex10-1*::pBHt2(hph) *zzz::pMG02 (URA5,* P_{TEF1}*-gfp:pts1)*
OSU281	*ura5-42Δ pex33-1::pBHt2(hph) zzz::pCR639 (URA5,* P_{TEF1}*-gfp)*
OSU282	*ura5-42Δ pex33-1::pBHt2(hph) zzz::pMG02 (URA5,* P_{TEF1}*-gfp:pts1)*
OSU341	*ura5-42Δ zzz::pAG21 (*$G418^R$*,GFP) zzz::pED02 (URA5, gfp-RNAi)*
OSU344	*ura5-42Δ zzz::pAG21 (*$G418^R$*,GFP) zzz::pQS34(URA5, gfp:SID1-RNAi)*
OSU377	*ura5-42Δ zzz::pQS01 (*$G418^R$*,* P_{TEF1}*-RFP:FLAG) pex11::pBHt2(hph)*
OSU389	*ura5-42Δ zzz::pQS01 (*$G418^R$*, RFP) zzz::pCR639 (URA5,* P_{TEF1}*-gfp)*
OSU390	*ura5-42Δ zzz::pQS01 (*$G418^R$*, RFP) zzz::pMG02 (URA5,* P_{TEF1}*-gfp:pts1)*
OSU391	*ura5-42Δ zzz::pQS01 (*$G418^R$*, RFP) pex5-1::pBHt2(hph) zzz::pCR639 (URA5,* P_{TEF1}*-gfp)*
OSU392	*ura5-42Δ zzz::pQS01 (*$G418^R$*, RFP) pex5-1::pBHt2(hph) zzz::pMG02 (URA5,* P_{TEF1}*-gfp:pts1)*
OSU393	*ura5-42Δ zzz::pQS01 (*$G418^R$*, RFP) pex11::pBHt2(hph) zzz::pCR639 (URA5,* P_{TEF1}*-gfp)*
OSU394	*ura5-42Δ zzz::pQS01 (*$G418^R$*, RFP) pex11::pBHt2(hph) zzz::pMG02 (URA5,* P_{TEF1}*-gfp:pts1)*
OSU399	*ura5-42Δ zzz::pAG21 (*$G418^R$*,GFP) zzz:pED02(URA5, gfp-RNAi)*
OSU400	*ura5-42Δ zzz::pAG21 (*$G418^R$*,GFP) zzz:pQS69(URA5, gfp:PEX7-RNAi)*
OSU449	*ura5-42Δ sid1-1Δ*
OSU483	*ura5-42Δ zzz::pAG21 (*$G418^R$*,GFP) zzz::pPB26 (URA5, gfp:PEX5-RNAi)*
OSU484	*ura5-42Δ zzz::pQS01 (*$G418^R$*, RFP:FLAG) pex11::pBHt2 (hph) zzz::pPB21 (URA5,* P_{PEX11}*-PEX11)*
OSU497	*ura5-42Δ sid1-1Δ zzz:pCS22(hph, td-tomato RFP:SID1)*

OSU517	*ura5-42Δ sid1-1Δ zzz:pCS11(hph, td-tomato RFP)*
OSU522	*ura5-42Δ zzz::pMG02 (URA5, P_{TEF1}-gfp:pts1) zzz::pCS12(hph, td-tomato RFP:SID3)*
OSU523	*ura5-42Δ zzz::pMG02 (URA5, PTEF1-gfp:pts1) zzz::pCS22(hph, td-tomato RFP:SID1)*
OSU545	*ura5-42Δ zzz::pMG02 (URA5, PTEF1-gfp:pts1) zzz::pCR865(hph, mTagBFP:HDEL)*
OSU553	*ura5-42Δ pex10-1::pBHt2(hph) zzz::pCR650 (URA5, P_{H2B}-PEX10) zzz::pPB35 ($G418^R$, P_{TEF1}-gfp:pts1)*
OSU554	*ura5-42Δ pex33-1::pBHt2(hph) zzz::pCR644 (URA5, P_{H2B}-PEX33) zzz::pPB35 ($G418^R$, P_{TEF1}-gfp:pts1)*
OSU555	*ura5-42Δ pex10-1::pBHt2(hph) zzz::pCR650 (URA5, P_{H2B}-PEX10) zzz::pPB36 ($G418^R$, td-tomato RFP:SID1)*
OSU556	*ura5-42Δ pex33-1::pBHt2(hph) zzz::pCR623 (URA5, P_{TEF1}-gfp) zzz::pPB36 ($G418^R$, td-tomato RFP:SID1)*
OSU557	*ura5-42Δ pex33-1::pBHt2(hph) zzz::pCR644 (URA5, P_{H2B}-PEX33) zzz::pPB36 ($G418^R$, td-tomato RFP:SID1)*
OSU565	*ura5-42Δ pex10-1::pBHt2(hph) zzz::pCR623 (URA5, P_{TEF1}-gfp) zzz::pPB36 ($G418^R$, td-tomato RFP:SID1)*

[1]all strains derived from the clinical isolate G217B (ATCC 26032).

[2]Gene designations:

BFP: blue fluorescence protein
$G418^R$: aminoglycoside phosphotransferase (G418 resistance)
GFP: green fluorescence protein
H2B: histone H2B
hph: hygromycin phosphotransferase (hygromycin B resistance)
PEX5: peroxisomal biogenesis factor 5
PEX7: peroxisomal biogenesis factor 7
PEX10: peroxisomal biogenesis factor 10
PEX11: peroxisomal biogenesis factor 11
PEX33: peroxisomal biogenesis factor 33
RFP: red-fluorescence protein
SID1: L-Ornithine N5-oxygenase
SID3: N(5)-hydroxyornithine:cis-anhydromevalonyl coenzyme A-N(5)-transacylase
TEF1: translation elongation factor EF-1 alpha
URA5: orotate phosphoribosyltransferase
